# Unravelling Main- and Side-Chain Motions in Polymers with NMR Spectroscopy and Relaxometry: The Case of Polyvinyl Butyral

**DOI:** 10.3390/polym13162686

**Published:** 2021-08-11

**Authors:** Lucia Calucci, Silvia Pizzanelli, Alessandro Mandoli, Artur Birczyński, Zdzisław T. Lalowicz, Cristina De Monte, Lucia Ricci, Simona Bronco

**Affiliations:** 1 Istituto di Chimica dei Composti OrganoMetallici, Consiglio Nazionale delle Ricerche—CNR, Via G. Moruzzi 1, 56124 Pisa, Italy; lucia.calucci@pi.iccom.cnr.it; 2 Centro per l’Integrazione della Strumentazione Scientifica dell’Università di Pisa (CISUP), Lungarno Pacinotti 43/44, 56126 Pisa, Italy; 3 Dipartimento di Chimica e Chimica Industriale, Università di Pisa, Via G. Moruzzi 13, 56124 Pisa, Italy; alessandro.mandoli@unipi.it; 4 Institute of Technology, The Pedagogical University of Kraków, Podchorążych 2, 30-084 Krakow, Poland; artur.birczynski@ifj.edu.pl; 5 Institute of Nuclear Physics, Polish Academy of Sciences, ul. Radzikowskiego 152, 31-342 Krakow, Poland; zdzislaw.lalowicz@ifj.edu.pl; 6 Istituto per i Processi Chimico-Fisici, Consiglio Nazionale delle Ricerche—CNR, Via G. Moruzzi 1, 56124 Pisa, Italy; cristina.demonte@pi.ipcf.cnr.it (C.D.M.); lucia.ricci@pi.ipcf.cnr.it (L.R.); simona.bronco@pi.ipcf.cnr.it (S.B.)

**Keywords:** glass transition, α-relaxation, secondary relaxation, ^2^H NMR, FID analysis, field cycling NMR relaxometry

## Abstract

Polyvinyl butyral (PVB) is an amorphous polymer employed in many technological applications. In order to highlight the relationships between macroscopic properties and dynamics at a microscopic level, motions of the main-chain and of the propyl side-chains were investigated between *T*_g_ − 288 °C and *T*_g_ + 55 °C, with *T*_g_ indicating the glass transition temperature. To this aim, a combination of solid state Nuclear Magnetic Resonance (NMR) methods was applied to two purposely synthesized PVB isotopomers: one fully protonated and the other perdeuterated on the side-chains. ^1^H time domain NMR and ^1^H field cycling NMR relaxometry experiments, performed across and above *T*_g_, revealed that the dynamics of the main-chain corresponds to the α-relaxation associated to the glass transition, which was previously characterized by dielectric spectroscopy. A faster secondary relaxation was observed for the first time and ascribed to side-chains. The geometry and rate of motions of the different groups in the side-chains were characterized below *T*_g_ by ^2^H NMR spectroscopy.

## 1. Introduction

Amorphous polymers find numerous applications in the nonwovens, adhesives, textiles, printing and packaging, paints and coatings, construction, and paper industries. Macroscopic thermal, rheological and mechanical properties allowing these applications are strongly connected to structural and dynamic properties of the polymers at a molecular level [1]. In particular, on increasing the temperature, polymers undergo a hierarchy of motional processes that cover wide length and time scales, spanning from very fast localized fluctuations and conformational isomerizations, to segmental dynamics, to collective chain motions up to free translational diffusion of the whole chain [2].

The most dramatic change in polymer properties is observed at the glass transition. Different dynamic processes are important for glass formation in amorphous polymers, as highlighted by a variety of methods such as mechanical-dynamical spectroscopy, light spectroscopy, neutron scattering, broad band Dielectric Spectroscopy (DS), Nuclear Magnetic Resonance (NMR) spectroscopy, and specific heat spectroscopy. The most prominent process is the so-called α-relaxation, also referred to as structural (primary) relaxation or dynamic glass transition, due to segmental fluctuations related to conformational changes in the polymeric main-chain. In the melt state well above the glass transition temperature (*T*_g_), the corresponding correlation time, *τ*_α_, has typical values of about 10^−13^ s, describing localized segmental fluctuations, also referred to as “glassy dynamics”; with decreasing temperature, the correlation time increases very rapidly, reaching typical values of ~100 s at *T*_g_. Most amorphous polymers show, in addition to the α-relaxation, secondary relaxations, named as β, γ, etc., due to local motions involving either pendant groups or main-chain units. A peculiar secondary relaxation, revealed by DS measurements on polymers without side-chains, is the Johari-Goldstein process, which has been considered as a generic feature of glass formation and can be regarded as a precursor for the glass transition [3]. On the other hand, in the case of polymers bearing side-chains, secondary relaxations associated to motions of the pendant groups were found [4,5,6,7,8]. Solid state NMR spectroscopy was revealed to be particularly useful in characterizing the mechanism of these motions [4,5].

Polyvinyl butyral (PVB) is an amorphous polymer widely used as an interlayer material in the manufacture of safety glass laminates, in solar photovoltaic modules, and as a binder in coatings, adhesives, enamels, and inks, thanks to its excellent optical clarity, adhesive properties, toughness, and flexibility [9,10,11,12]. The amorphous character of PVB arises from the random distribution of vinyl butyral and vinyl alcohol units, as well as from the presence of propyl side-chains on vinyl butyral units.

**Figure 1:**
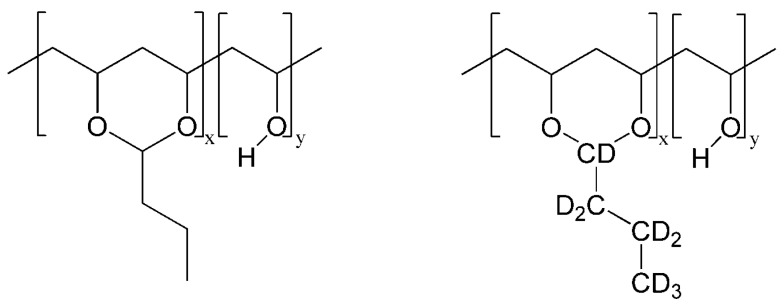
Structure of PVB-H and PVB-D. Fractions of vinyl butyral, x, and vinyl alcohol, y, are 0.6 and 0.4, respectively (see Supplementary Materials).

In spite of the large number of technological applications, only a few studies were reported concerning the dynamics of PVB [13,14,15,16,17,18,19,20,21,22]. Recently, PVB dynamics was investigated by some of us over broad frequency and temperature ranges by DS and ^1^H field-cycling (FC) NMR relaxometry, focusing on a commercial sample [21]. DS allowed the α-relaxation to be characterized. Correlation times for this dynamic process (*τ*_α_) were determined as a function of temperature, from which a dynamic glass transition temperature and a fragility index were extracted. Below *T*_g_, a β-relaxation was found, and the corresponding correlation times (*τ*_β_) were determined. ^1^H FC NMR, a technique measuring the dependence (dispersion) of proton longitudinal relaxation rate (*R*_1_) on the Larmor frequency (vide infra), was applied to study glassy dynamics above *T*_g_ up to *T*_g_ + 50 °C. The evolution of the *R*_1_ dispersion curves with temperature indicated dynamic heterogeneity in proximity of the glass transition. Indeed, different dynamic properties can be envisaged for different segments of PVB considering that its structure includes a main-chain made of rigid units and quite long and more mobile alkyl side-chains (Figure 1).

In order to better characterize the dynamics of PVB in the glassy and rubbery state and, in particular, to unravel the dynamics of side- and main-chains, in the present work complementary NMR techniques were applied to two purposely synthesized PVB samples: PVB-H, fully protonated, and PVB-D, perdeuterated on the side-chains (Figure 1). In particular, the dynamics of the side-chains below *T*_g_ was investigated by ^2^H NMR spectroscopy on PVB-D. The line shape of ^2^H NMR spectra is determined by the modulation of the dominant quadrupolar interaction of ^2^H nuclei by molecular motions, and information on the rate and mechanism of motion can be obtained from spectral line shape analysis [23,24]. In protonated moieties, the decay in time of the ^1^H NMR signal (free induction decay, FID) is dominated by the effects of ^1^H-^1^H dipole-dipole couplings fluctuating because of molecular motions. Therefore, a strong connection exists between molecular mobility and ^1^H time domain signals, with more mobile systems showing a more slowly decaying signal. Information on polymer dynamics can thus be obtained by FID analysis [25,26,27]. Here, FID’s of PVB-H and PVB-D were analyzed at different temperatures to track the dynamics, mainly associated to the α-relaxation, across the glass transition. Finally, ^1^H FC NMR relaxometry was applied to both PVB-H and PVB-D above *T*_g_ to more quantitatively characterize the dynamics of the different moieties. In fact, proton longitudinal relaxation results from the modulation of ^1^H-^1^H intra- and intermolecular dipolar interactions by polymer motions [28]. The possibility of measuring *R*_1_ over a wide frequency range (0.01 to 40 MHz with commercial relaxometers) renders ^1^H FC NMR relaxometry a powerful technique for investigating polymer dynamics. Here, experiments were performed up to 120 °C, corresponding to *T*_g_ + 55 °C. In the available frequency and temperature ranges, ^1^H FC NMR relaxometry allowed glassy dynamics and propyl side-chain isomerizations to be investigated and correlation times to be estimated for these motions at the highest temperature. All together the applied techniques gave a picture of side- and main-chain motions over a quite broad temperature range, spanning from −223 °C to 120 °C, that is from *T*_g_ − 288 °C to *T*_g_ + 55 °C.

## 2. Materials and Methods

### 2.1. Materials

**Scheme 1:**
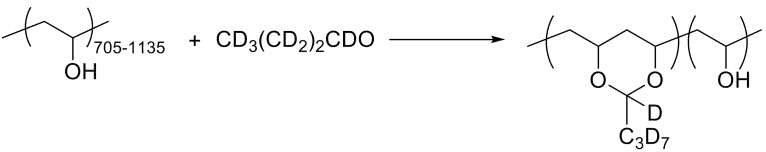
Preparation of the deuterated polyvinyl butyral PVB-D.

PVB-D was synthesized starting from polyvinyl alcohol (*M*_w_ = 31–50 kg/mol, 98–99% hydrolyzed) and butanal-*d*_8_ according to Scheme 1 [29]. Butanal-*d*_8_ was prepared oxidizing butanol-*d*_10_ with Dess-Martin periodinane, following Ref. [30]. The deuteration degree of PVB-D is >95%. PVB-H was synthesized adopting the same strategy but using polyvinyl alcohol (*M*_w_ = 31–50 kg/mol, 98–99% hydrolyzed) and butanal. Details of the synthetic procedures are reported in the Supporting Information. PVB-H and PVB-D were characterized by a molecular weight, *M*_w_, of 101 and 106 kg/mol and a polydispersity of 1.6 and 2.0, respectively, as determined by size exclusion chromatography. *T*_g_ values, determined by DSC, were 65.6 °C for PVB-H and 64.0 °C for PVB-D. The molar fractions of vinyl butyral and vinyl alcohol units in PVB-H, determined according to Ref. [29] from ^1^H NMR in CDCl_3_, are 0.6 and 0.4, respectively.

### 2.2. Differential Scanning Calorimetry (DSC) and Size Exclusion Chromatography (SEC) Measurements

DSC experiments were performed using a Seiko SII ExtarDSC7020 calorimeter (Chiba, Japan) with the following thermal protocol: first cooling from 20 to 0 °C; at 0 °C for 2 min; first heating from 0 to 110 °C; 110 °C for 2 min; second cooling from 110 to 0 °C; at 0 °C for 2 min; second heating from 0 to 110 °C; at 110 °C for 2 min; third cooling from 110 to 20 °C. The cooling/heating rate was 10 °C/min in all runs, except for the third cooling, for which it was 30 °C/min. About 5 mg of polymer were used for each measurement. The *T*_g_ was determined using the tangents to the measured heat capacities below and above the heat capacity step via the Muse TA Rheo System software (version 3.0).

Molecular weight (*M*_w_) and polydispersity were determined by SEC using an Agilent Technologies 1200 Series instrument equipped with two PLgel 5 mm MiniMIX-D columns (flux 0.3 mL/min) and a refraction index detector. Monodisperse poly(styrene) samples were used as calibration standards. The analysis was performed on a PVB solution in chloroform (2 mg/mL) filtered through a 0.2 mm filter prior to the measurement.

### 2.3. NMR Measurements

^2^H NMR spectra were recorded at 46 MHz on a spectrometer constituted by a Magnex superconducting magnet and a Tecmag Apollo 500 NMR console and equipped with a probe mounted inside the Oxford Instruments CF1200 continuous flow cryostat. Measurements were performed between −223 and 20 °C. The temperature was regulated by the Oxford Instruments CT503 Temperature Controller to the accuracy of ±0.1 °C. The quadrupole echo pulse sequence [31] was applied with appropriate phase cycling, with a 90° pulse duration of 5 μs and a time interval between the pulses of 50 μs. The spectra were obtained after Fourier Transformation of the signal collected from the echo maximum. The recycle delay ranged between 2 and 300 s depending on temperature. For the ^2^H NMR measurements, the samples (200–300 mg) were enclosed in 5 mm o.d. glass tubes of 20 mm length that fitted the probe coil and sealed under vacuum.

Time domain ^1^H NMR experiments were performed at a Larmor frequency of 20.7 MHz using a Niumag permanent magnet interfaced with a Stelar (Mede, Italy) PC-NMR console. Samples (100–200 mg) were enclosed in 5 mm o.d. NMR glass tubes, which were evacuated and flame sealed. ^1^H FIDs were recorded on-resonance and in full-absorption mode receiver setting using both the direct excitation and the solid echo [32] pulse sequences with a 90° pulse duration of 3 μs and a recycle delay of 3 s and accumulating 128 transients. For the solid echo experiments, an echo delay of 14 μs was employed. Spectra were recorded at different temperatures between 30 and 100 °C. The temperature was controlled within ±0.2 °C by a Stelar VTC90 variable temperature controller and the sample was allowed to equilibrate for at least 10 min before measurement. The signal intensity was corrected according to the Curie law by multiplication with *T*/*T*_ref_, with *T*_ref_ = 373 K. FID data up to 0.2 ms were considered in the analysis because the signal decay at longer times is affected by field inhomogeneities.

^1^H longitudinal relaxation times (*T*_1_) were measured at different Larmor frequencies using a Spinmaster FFC-2000 relaxometer (Stelar, Mede, Italy). The experiments were performed applying the prepolarizing and non-prepolarizing pulse sequences [33,34] below and above 10 MHz, respectively. The polarizing frequency was 25 MHz and the detection frequency 16.3 MHz. The 90° pulse duration was 10 μs and the switching time 3 ms. Four transients were accumulated and at least 16 values of the variable delay were used to build the magnetization trends. All the other parameters were optimized for each experiment. Samples (100–200 mg) were enclosed in 10 mm o.d. NMR glass tubes, which were evacuated and flame sealed. Measurements were performed at different temperatures in the 95 to 120 °C range, letting the sample temperature equilibrate for at least 10 min. The sample temperature was controlled within ±0.2 °C by a Stelar VTC90 unit.

## 3. Results and Discussion

### 3.1. Dynamics of Side-Chains below T_g_ by ^2^H NMR Spectroscopy

**Figure 2:**
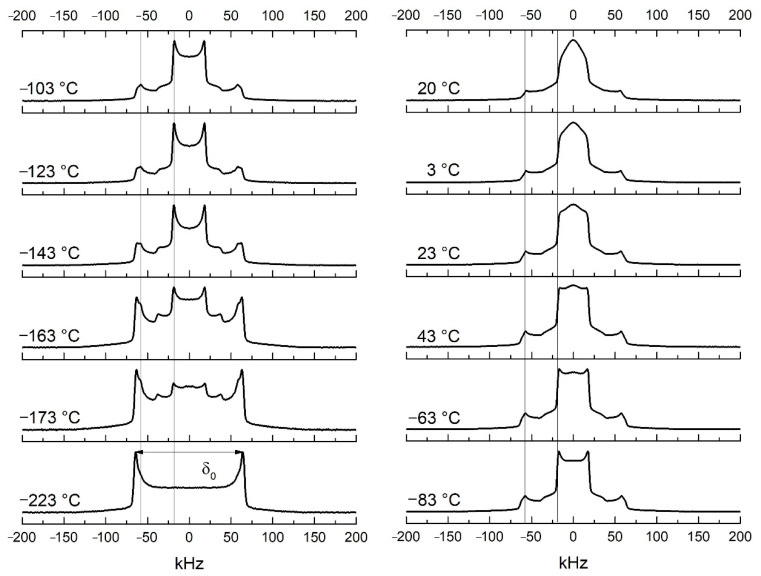
^2^H NMR spectra of PVB-D at the indicated temperatures. Vertical lines are guides for the eyes. The spectra were normalized to a constant height.

^2^H NMR spectra were acquired on PVB-D at several temperatures between −223 °C and 20 °C to investigate the dynamics of the side-chains from the rigid limit up to *T*_g_ − 45 °C; a selection of spectra is shown in Figure 2. All the spectra result from the superposition of ^2^H NMR subspectra relative to the different side-chain groups in PVB-D. In turn, each subspectrum is given by the sum of signals from all possible orientations of the C-D bonds in the amorphous sample (powder spectrum). In general, in a rigid powder spectrum one can observe inner singularities split by $2V_{yy}=\delta_{0}\left(1-\eta_{0}\right)$, inner edges separated by $2V_{xx}=\delta_{0}\left(1+\eta_{0}\right)$, and a total width of $2V_{zz}=2\delta_{0}$. *δ*_0_ and *η*_0_ are the anisotropy and asymmetry parameters related to the ^2^H electric field gradient tensor components and the quadrupolar coupling constant by the relationships

(1) $$\delta_{0}=\frac{3e^{2}qQ}{4\hbar }=\frac{3eQ}{4\hbar }V_{zz}$$

(2) $$\eta_{0}=\frac{V_{xx}-V_{yy}}{V_{zz}}$$

The asymmetry parameter *η*_0_ is usually negligible in aliphatic C-D bonds. Therefore, when *η*_0_ = 0, the inner singularities and edges converge, leading to strong singularities. This situation is observed at the lowest temperature for PVB-D (−223 °C, Figure 2), where a symmetric powder pattern is recorded, characterized by the typical quadrupolar splitting between inner singularities for aliphatic C-D bonds of 128 kHz ($=2V_{xx}=2V_{yy}=\delta_{0}$) [35,36]. The spectral edges at ± 128 kHz corresponding to ± *V_zz_* are smoothed due to finite pulse width [37].

When C-D bonds undergo a rapid motion, characterized by a rate much greater than *δ*_0_, an “averaged” spectrum results with singularities corresponding to averaged *δ* and *η* parameters, which depend on the motion geometry. For instance, for a methyl group rotating fast about its C3 axis, with deuterons subject to fast exchange between three equivalent sites of tetrahedral geometry, the singularities are expected to be split by $2V_{xx}=2V_{yy}=\delta_{0}/3$ and $2V_{zz}=2\delta_{0}/3.$ If the motional rate is on the order of *δ*_0_ (intermediate regime), the spectral line shape reflects both the rate and geometry of the motion. All this considered, the ^2^H NMR spectrum at −173 °C can be interpreted as the superposition of a rigid component ascribable to methine (CD) and methylene (CD_2_) groups and a narrower pattern, showing singularities at about ±18 kHz and ±37 kHz, due to the methyl group (CD_3_). In particular, the shape of the latter component is compatible with that of a rotating methyl group in the intermediate regime. Indeed, the experimental spectral features are captured by a simulation including methyl deuterons undergoing three-site jumps with a rate of 4000 kHz and both rigid and mobile methine and methylene deuterons (Figure S4a). Indeed, the features observed at ±59 kHz (see vertical line at −59 kHz in Figure 2) indicate that at −173 °C, a fraction (~40%) of CD and CD_2_ groups is not rigid anymore. At −123 °C, the rigid pattern has disappeared, and an averaged spectrum is observed for the CD and CD_2_ groups characterized by singularities at ±58 kHz and inner edges at ±63 kHz (Figure 2), resulting in δ/δ_0_ = 0.946 and η = 0.044. Similar line shapes were reported for deuterated methylene groups in the main-chain of poly(ethyl methacrylate) and poly(methyl methacrylate) [38] and of poly(ethylene-*alt*-propylene) [39] at temperatures below *T*_g_ and for methylene groups in the side-chains of poly(diethylsiloxane) in a crystalline phase [40]. This pattern can be interpreted as due to C-D bonds subject to a fast two-site jump motion in a cone, with cone angle of 70.5°, typical of tetrahedral symmetry, and flip angle of ~20° [40]. Upon heating, the pattern persists up to room temperature, but it slightly narrows and shows a progressively reduced asymmetry, as indicated by the values of δ/δ_0_ and η extracted from the spectra and reported in Figure S5. The trends observed for these parameters cannot be rationalized by a simple increase of the flip angle, which would give a decrease of δ/δ_0_ but an increase of η; instead, a motion more complicated than a two-site jump should be invoked, possibly involving a reorientation of the cone axis itself by a small flip angle. As a matter of fact, a fast motion characterized by small amplitudes is suggested by the modest narrowing of the pattern upon heating from −173 °C up to room temperature. As far as methyl is concerned, between −143 and −123 °C, the corresponding line shape shows the pattern expected for the fast rotation about the C3 axis (Figure 2), with a decreased signal intensity in the region between the ±18 kHz singularities. Indeed, at −123 °C, the ^2^H NMR spectrum can be satisfactorily reproduced by a combination of two subspectra, one due to CD_3_ groups rotating fast about their ternary axes and the other arising from CD and CD_2_ moieties undergoing fast two-site jumps (Figure S4b). Upon heating up to room temperature, the methyl signal intensity increases in the central part of the spectrum, and there is a partial collapse of the singularities at ±18 kHz (Figure 2); these features are indicative of the onset of a motion of the methyl axis further averaging the residual quadrupolar interaction [41,42,43]. All in all, at 20 °C (=*T*_g_ − 45 °C), the highest temperature we could set in our instrument, the side-chains are still subjected to small amplitude motions. At this temperature, the CD_2_ and CD groups experience a fast spatially restricted motion, whereas the methyl group undergoes both a fast motion about its C3 axis and a slower motion involving the C3 axis itself. This motion is not isotropic yet and occurs at rates on the order of the residual quadrupolar interaction.

Further information on the dynamics of the PVB side-chains arises from the total intensity of the ^2^H NMR spectra as a function of temperature, as well as from the relative intensity of the spectral contributions due to the different groups. Indeed, distortions in the line shape and significant loss of signal intensity can be observed in ^2^H NMR spectra acquired with the quadrupolar echo pulse sequence when motions of the C-D bonds occur in the so-called intermediate regime, i.e., when the motional rates are of the order of δ_0_ [31], while insignificant and little intensity loss is expected for motions in the slow and fast regime, respectively [41,42,43,44,45]. Moreover, spectral distortions and signal loss depend on the echo delay and on the motion geometry. In particular, it has been shown that spatially restricted motions with correlation times on the order of the quadrupolar echo time, as in the case for secondary relaxation processes in glasses, lead to strong loss of the spectral intensity for echo times longer than few tens μs [46,47,48].

**Figure 3:**
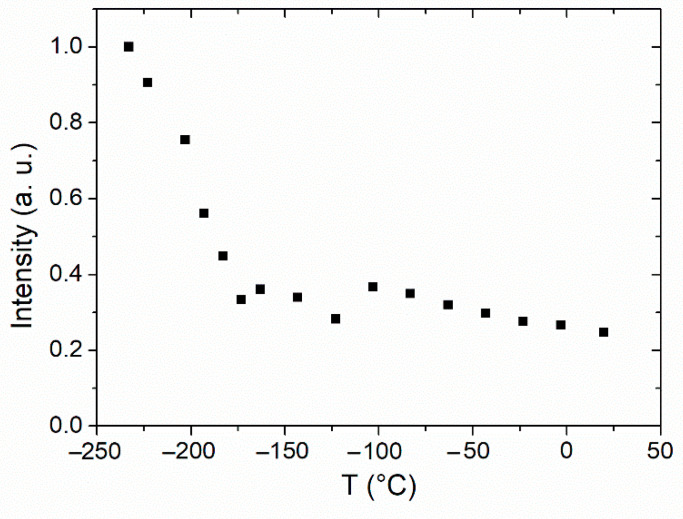
Integral signal intensity of ^2^H NMR spectra of PVB-D collected with a solid echo delay of 50 μs at various temperatures. The intensities were divided by the intensity of the spectrum at −223 °C.

The total integrated signal intensity of the ^2^H spectra recorded on PVB-D at various temperatures reported in Figure 3 shows a rapid and pronounced decrease from −223 to −173 °C, while at higher temperatures a less steep decrease is observed. The signal loss is associated to the presence of motions in the intermediate regime. Therefore, in the recorded spectra, the contributions of groups undergoing slow and fast motions are enhanced, although a distribution of motional rates is probably present. At −173 °C, the spectral contributions from CD_3_ moieties (0.37) and from CD_2_ and CD groups (0.63), as reported in Figure S4a, are close to the value expected on the basis of the chemical structure of the side chain (0.30 and 0.70, respectively). Therefore, motions in the intermediate regime affect all the propyl chain groups. At higher temperatures, by looking at the evolution of the relative intensities of the outer and inner parts of the spectra in Figure 2, mainly ascribed to CD and CD_2_ groups and to CD_3_ groups, respectively, a collapse is observed for the intensity of the outer part with respect to that of the inner part at −143 °C. Then the relative intensity of the outer part of the spectra only slightly decreases by further increasing the temperature up to 20 °C. The simulation of the line shape at −123 °C indicates a spectral contribution from CD_2_ and CD of only 0.24, paralleled by a CD_3_ contribution of 0.76. Similarly, a selective signal loss of methine and methylene groups with respect to methyl groups was observed below *T*_g_ in poly(ethylene-*alt*-propylene) [39]. This feature was ascribed to the different behavior of C-D bonds in methine and methylene groups undergoing restricted angular displacements (i.e., wobbling in a cone with an angle of 11°−31°, depending on the temperature) in contrast to that of methyl groups, which also show fast rotation about their C3 axis. In our case, the reorientation in a cone for the CD_2_ and CD groups, with a flip angle of ∽20°, results in a significant loss of their signal compared to that of CD_3_.

### 3.2. Dynamics of Main- and Side-Chains across T_g_ from ^1^H FID Analysis

**Figure 4:**
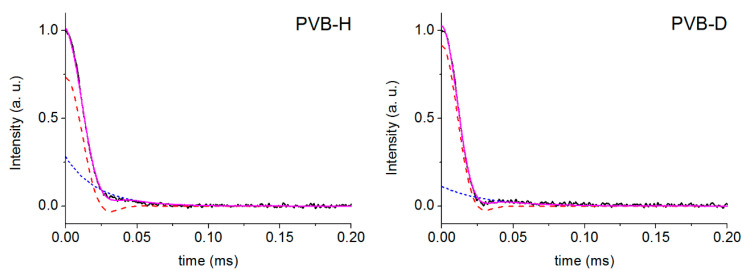
Analysis of the ^1^H FIDs (black full lines), recorded by the solid echo pulse sequence on PVB-H (**left**) and PVB-D (**right**) at 30 °C, as a sum (purple full lines) of an Abragamian function (red dashed lines) and an exponential function (blue short-dashed lines).

**Figure 5:**
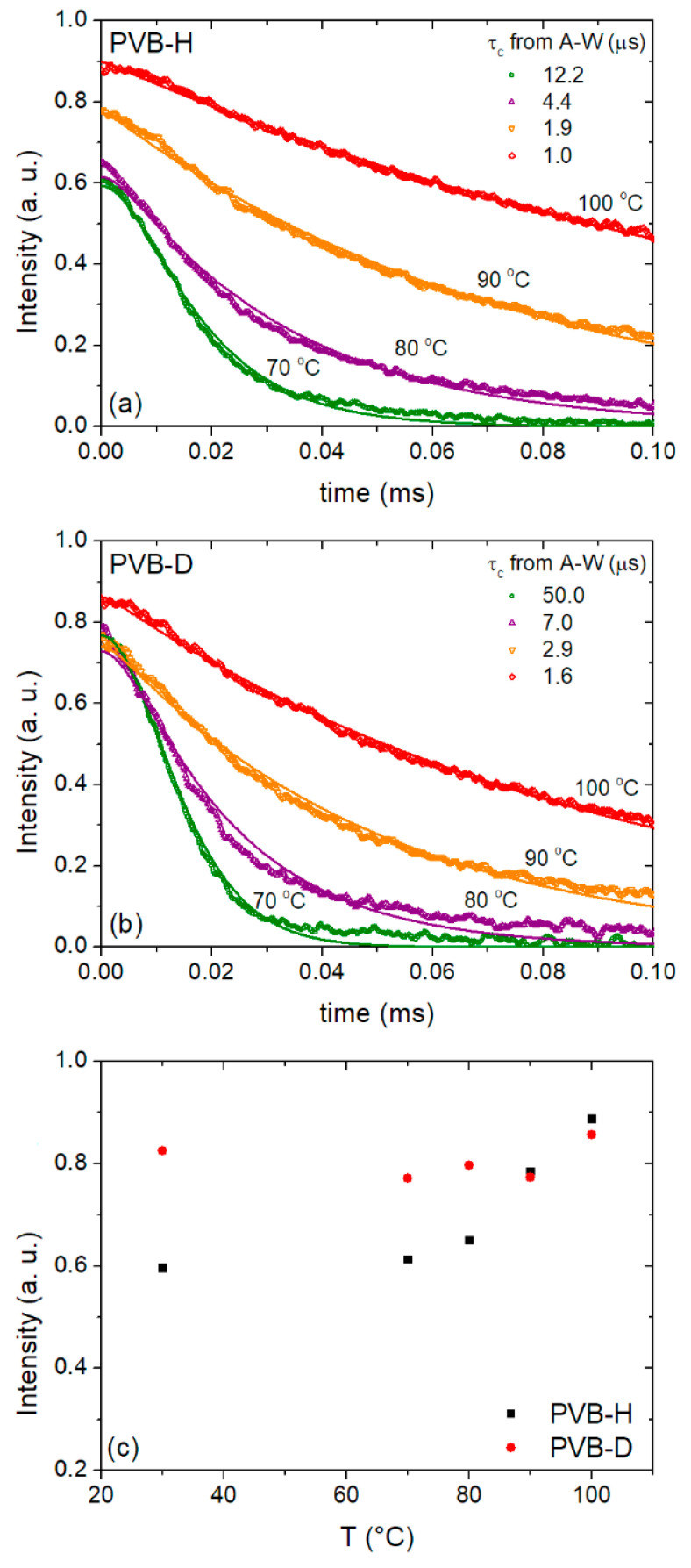
^1^H FIDs recorded on PVB-H (**a**) and PVB-D (**b**) at the indicated temperatures with the solid echo pulse sequence (symbols) and fitting curves to Equation (4) (solid lines). (**c**) Intensity of the signal at the top of the echo divided by the intensity of the FID at *t* = 0 recorded by direct excitation at the reference temperature (100 °C). FID intensities were corrected according to the Curie law.

In order to gain information on the dynamics of main- and side-chain segments across *T*_g_, the ^1^H FIDs of PVB-H and PVB-D were acquired between 30 and 100 °C, corresponding to temperatures of *T*_g_ − 35 °C and *T*_g_ + 35 °C, using both the solid echo and the direct excitation pulse sequences; data collected using these sequences are shown in Figure 4, Figure 5 and Figure S6. The observed signals arise from both main- and side-chain hydrogens for PVB-H, but from the sole main-chain hydrogens for PVB-D. The polymer mobility can be investigated by studying the FID signal, a fast decay of the signal corresponding to the response of the rigid glassy polymer segments and a slower one to that of mobile segments. However, the FID acquired by direct excitation (i.e., the decays of the transverse magnetization after a 90° pulse) cannot be directly exploited to obtain the signal arising from polymer segments in the glassy state showing significant decay within 20 μs. In fact, the first 14 μs of the FID, containing the initial fast decaying part of the signal, are missing because of the dead time of the spectrometer receiver. An accurate measurement of the shape of the initial part of the FID for a rigid phase can instead be obtained with the solid echo pulse sequence because it avoids the dead time of the spectrometer. However, the amplitude of the echo signal can be lower than its true value because the solid echo does not fully refocus the multi-spin dipolar interactions present in a proton dipolar network. Moreover, further signal loss may occur for polymer segments undergoing motions with correlation times comparable to the echo time. All this considered, in our analysis we adopted the following strategy: (i) the solid echo FIDs were used to obtain information on the shape of the signal decay for PVB-H and PVB-D, and therefore on the polymer dynamics; (ii) the FIDs recorded by direct excitation were exploited to quantitatively determine the signal intensity arising from all protons in the polymers [49,50]; (iii) the comparison of the echo amplitude at maximum with the reconstructed intensity at *t* = 0 of the direct excitation FID was employed for quantifying the signal losses due to polymer dynamics on a time scale on the order of the echo delay (10–100 μs).

In the glassy state at 30 °C, for both PVB isotopomers, most of the signal decays were within 25 μs (Figure 4), indicating that almost all the hydrogens are in rigid environments with characteristic motion frequencies smaller than tens of kHz on the order of the static ^1^H-^1^H dipolar couplings. The remaining hydrogens contribute to a more slowly decaying FID component, which is larger for PVB-H than for PVB-D. In the case of PVB-D, the fast-decaying part of the FID shows a “hole” at about 30 μs, typical of a Pake profile, arising from the dominant dipolar coupling between ^1^H nuclei in CH_2_ groups in the vinyl butyral and vinyl alcohol units. For PVB-H, the Pake “hole” is smeared out, most probably because of the presence of a larger number of ^1^H-^1^H pairs with different dipolar couplings. For both PVB-D and PVB-H, the FIDs at 30 °C were fitted to the sum of an Abragamian function and an exponential function:

(3) $$I\left(t\right)=w_{Abr}\frac{sin\left(2\pi \nu t\right)}{2\pi \nu t}e^{-{\left(t/T_{2,Abr}\right)}^{2}}+w_{exp}e^{-(t/T_{2,exp})}$$

The Abragamian function is a Gaussian broadened *sinc* function introduced by Abragam [51], which is generally used to reproduce the signal decay associated to a Pake spectrum. In Equation (3), *w_i_*, with *i* = *Abr*, *exp*, represents the weight fraction of hydrogen nuclei in domain *i* and *T*_2,*i*_ the corresponding time constant. A good reproduction of the FIDs (see Figure 4) was obtained with *ν* = 19922 s^−1^ for both isotopomers and with *T*_2,*Abr*_ = 26 μs, *w_exp_* = 0.28 and *T*_2,exp_ = 22 μs for PVB-H and *T*_2,*Abr*_ = 22 μs, *w_exp_* = 0.11 and *T*_2,exp_ = 30 μs for PVB-D. The small *T*_2_ values associated to both components indicate that slow and/or amplitude restricted dynamics occur in both PVB-D and PVB-H at *T*_g_ − 35 °C. Second moment values, *M*_2_, of 6.7·10^9^ and 7.3·10^9^ rad^2^ s^−2^ are associated to the main Abragamian component for PVB-H and PVB-D, respectively.

Upon increasing the temperature up to *T*_g_ + 35 °C, the evolution of the signal reflects the motional narrowing phenomenon associated to the glass-rubber transition (Figure 5 and Figure S6). A comparison between FIDs acquired at the same temperature for the two isotopomers shows a slower signal decay for PVB-H with respect to PVB-D at all the temperatures, indicating faster dynamics. In order to extract semi-quantitative information, we used the Anderson–Weiss (A-W) model [52] to analytically express the FID of a dipolarly coupled spin pair subject to isotropic rotational diffusion:

(4) $$I\left(t\right)\approx exp\left[-M_{2}\tau_{c}^{2}\left(e^{-t/\tau_{c}}+\frac{t}{\tau_{c}}-1\right)\right]$$

where the second moment, *M*_2_, is approximately equal to $\left(9/20\right)D_{HH}^{2}$, with $D_{HH}$ representing the largest dipole–dipole pair coupling. *τ_c_* indicates the correlation time associated to rotational diffusion. Here, *τ_c_* represents the correlation time for the main-chain segmental reorientations (α-process) for PVB-D, whereas it non-trivially results from a combination of main-chain and faster side-chain motions for PVB-H. The A-W model was previously applied to analyze the FID signals of poly(ethyl acrylate) around and in particular above *T*_g_ [49]. This model satisfactorily fits the experimental signal decays of PVB-H and PVB-D, obtained by both solid echo (Figure 5) and direct excitation (Figure S6) pulse sequences, with *M*_2_ taken from the fit to the Abragamian function and *τ_c_* values as reported in the figures. Between 70 and 100 °C, *τ_c_* decreases from tens of microseconds to approximately one microsecond, with small differences between values extracted from signals acquired by the two techniques. PVB-D shows longer *τ_c_* values compared to PVB-H at the same temperature. Therefore, main-chain segmental fluctuations associated to the α-process, selectively detected in PVB-D, are slower than the combined dynamic process revealed in PVB-H, where faster side-chain motions affect the observed *τ_c_*.

Further information on the dynamic behavior of PVB-D and PVB-H was obtained by quantifying the signal losses in solid echo experiments. To this aim, the signal intensities at the top of the solid echoes were corrected according to the Curie law and divided by the value at *t* = 0 of the direct excitation signal at the reference temperature (100 °C). In turn, the latter intensity was obtained from the fit of the signal to the A-W equation. The obtained results are shown in Figure 5c. Both samples exhibit signal losses at all the investigated temperatures. These losses are due to both unrefocused multi-spin ^1^H-^1^H dipolar interactions by the solid echo pulse sequence and molecular motions taking place during the echo delay in the intermediate regime, i.e., on the time scale of the refocused dipolar interaction (10–100 kHz). The larger signal loss observed for PVB-H with respect to PVB-D up to 80 °C can be ascribed to the larger number of multi-spin interactions present in PVB-H due to the larger amount of neighboring protons in this isotopomer. Starting from 80 °C, a faster recovery of the signal intensity is observed for PVB-H, most probably because many segments are undergoing fast motions with correlation times much shorter than 10 μs, especially in the side-chains.

### 3.3. Dynamics of Main- and Side-Chains above T_g_ from ^1^H FC NMR Relaxometry

**Figure 6:**
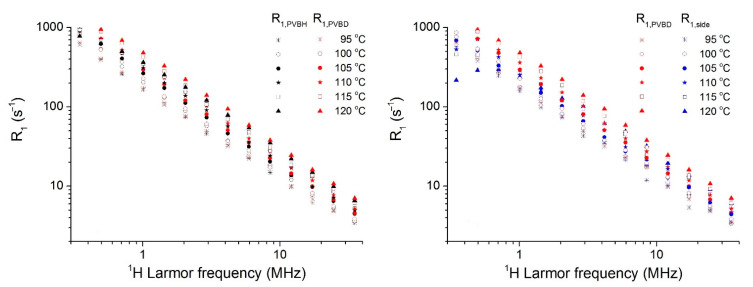
^1^H *R*_1_ of PVB-H (black symbols) and PVB-D (red symbols) and *R*_1,*side*_ (blue symbols) as a function of Larmor frequency at the indicated temperatures. *R*_1,*side*_ values were obtained using Equation (5).

**Figure 7:**
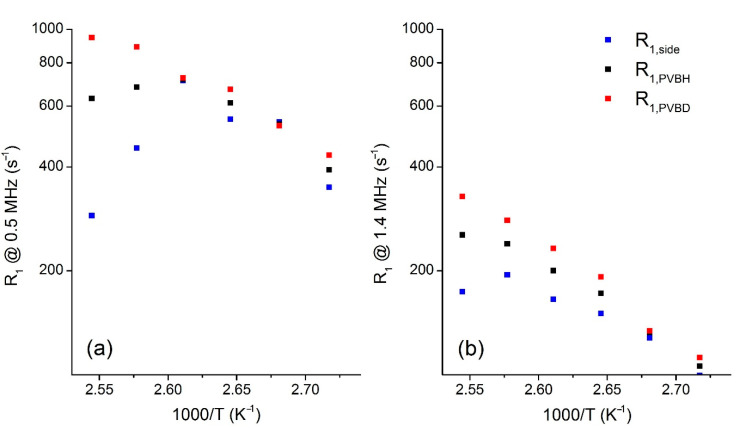
(**a**) ^1^H *R*_1_ of PVB-H and PVB-D and *R*_1,*side*_ at 0.5 MHz as a function of inverse temperature. (**b**) ^1^H *R*_1_ of PVB-H and PVB-D and *R*_1,*side*_ at 1.4 MHz as a function of inverse temperature.

^1^H longitudinal relaxation rates (*R*_1_ = 1/*T*_1_) were measured as a function of Larmor frequency between 0.3 and 35 MHz on both PVB-H and PVB-D at several temperatures above *T*_g_ (95–120 °C) in order to get insight into the dynamics of the main- and side-chains over a broad frequency range. At all frequencies and temperatures, a single *R*_1_ value was measured, indicating that spin diffusion is effective. *R*_1_ values could not be measured at lower frequencies because of limitations due to the relaxometer switching time. As shown in Figure 6, for PVB-D *R*_1_ values increase by increasing the temperature, indicating that for temperatures up to *T*_g_ + 55 °C motions of the main-chain segments are in the slow regime. Considering the frequency range observed, we can infer that dynamics occurs with a correlation time *τ* > 5∙10^−7^ s at 120 °C. On the other hand, an increasing trend of *R*_1_ with temperature is observed for PVB-H at frequencies above 1 MHz, while *R*_1_ reaches a maximum and then decreases at lower frequencies for *T* > 105 °C. This behavior is exemplified in Figure 7a, where the trend of *R*_1_ as a function of the inverse temperature is shown at the Larmor frequency of 0.5 MHz. A maximum of *R*_1_ is found at 110 °C, indicating that the motional frequency matches the measuring frequency. An estimate of the correlation time for the motion governing relaxation in the observed frequency range can be obtained by the relationship at the maximum of *R*_1_, ωτ≅1; a value of *τ* of ~3∙10^−7^ s is determined. This value is in agreement with that previously determined for commercial PVB at the same temperature [21].

If *R*_1_ data recorded at the same temperature on the two isotopomers are compared, lower values are observed at each frequency for PVB-H with respect to PVB-D. This feature is surprising if we consider the higher number of proton dipolar couplings contributing to relaxation in PVB-H with respect to PVB-D. In fact, in a polymer, each proton relaxes with a rate given by the sum of the dipolar interactions with all the other protons (interactions with deuterons in the case of PVB-D are negligible), weighted by *r*^−6^, *r* being the distance between proton pairs [53,54]. As a consequence, the relaxation strength should decrease in the deuterated sample, as previously observed for selectively deuterated polyisoprene [55]. The observed feature can instead be accounted for by considering that, due to spin diffusion, in PVB-H *R*_1_ represents an average over both side- and main-chain protons, whereas in PVB-D, only the main-chain protons contribute to relaxation. Assuming that PVB-D and PVB-H have the same fractions of vinyl butyral and vinyl alcohol units and that the main-chain dynamics does not change at a given temperature for the two isotopomers (*M*_w_ and *T*_g_ are quite similar), we can express the relaxation rate of PVB-H, *R*_1,*PVBH*_, as the average of the relaxation rate of PVB-D, *R*_1,*PVBD*_, due to main-chain protons, and that for the side-chain protons, *R*_1,*side*_, weighted by the molar fractions of hydrogens in the main- (*f*_*main*_ = 0.52) and side- (*f*_*side*_ = 0.48) chains arising from the stoichiometric composition of PVB-H:

(5) $$R_{1,PVBH}=f_{main}R_{1,PVBD}+f_{side}R_{1,side}$$

Another assumption of this approach is that, based on distance considerations, the observed *R*_1_ values are dominated by the fluctuations of dipolar interactions between two protons bound to the same carbon atoms and thus both belonging either to the main-chain or to a side-chain, while only minor contributions arise from dipolar interactions between a proton located on a side-chain and one located on the main-chain. Using Equation (5), *R*_1,*side*_ was determined at each temperature and frequency starting from the experimental relaxation rates. The obtained *R*_1,*side*_ values, shown in Figure 6, are always smaller than the corresponding *R*_1,*PVBD*_ values, the difference between them increasing with decreasing the frequency and increasing the temperature. At 120 °C, *R*_1,*side*_ shows a plateau below 1 MHz, indicating that side-chain dynamics has entered the motional narrowing regime. Looking at the relaxation rates as a function of temperature, maxima are observed for *R*_1,*side*_ at frequencies ≤1.4 MHz. In particular, at 0.5 MHz, the *R*_1,*side*_ maximum occurs at ~110 °C as for PVB-H (Figure 7b). Therefore, the maximum detected for PVB-H can be ascribed to the side-chain motion. At 1.4 MHz, the *R*_1,*side*_ maximum is found at ~115 °C (Figure 7a); correspondingly, a value of ~1.1∙10^−7^ s is determined for the correlation time of the side-chain motion.

The interpretation of the FC NMR data confirms the results of the ^1^H FID analysis concerning the faster dynamics of the side-chains compared to the main-chain. It also provides an estimate for the correlation time of the side-chain motion, i.e., ~1.1∙10^−7^ s at 115 °C. Moreover, a lower boundary of 5∙10^−7^ s can be set for the correlation time of the main-chain at 120 °C, the highest investigated temperature. These values are in line with those estimated by the Anderson–Weiss analysis of the ^1^H FIDs up to 100 °C.

If we compare these results with our previous findings on commercial PVB [21], we can state that the estimated value for the correlation time of the main-chain motion agrees with that determined for the α-relaxation by DS measurements. On the other hand, the correlation time of the side-chain motion at 115 °C is much longer than that associated to the β-relaxation as extrapolated at the same temperature from DS data. As a matter of fact, the dynamics of the propyl side-chains, associated to a very small variation of the electric dipole, is likely not to significantly affect dielectric relaxation.

## 4. Conclusions

Three NMR spectroscopy and relaxometry methods were applied to investigate the dynamics of the propyl side-chains and of the main-chain of PVB over a wide temperature range, spanning from −223 to 120 °C (i.e., from *T*_g_ − 288 °C to *T*_g_ + 55 °C), exploiting the PVB-D and PVB-H isotopomers. For the side-chains at temperatures lower than *T*_g_, ^2^H NMR experiments revealed heterogeneous dynamics for all the groups, with a significant amount of groups undergoing motions in the intermediate regime (10^4^–10^6^ Hz), leading to significant signal losses starting from −173 °C. The analysis of the line shapes of ^2^H NMR spectra indicates that the methine, methylene, and methyl groups undergo motions characterized by frequencies smaller than 10^5^ Hz up to –173 °C. At this temperature, the methyl groups are subjected to a three-site jump motion around the C3 axis with a rate of 4·10^6^ Hz, while a small fraction of CD and CD_2_ groups undergo a two-site jump motion in a cone with a flip angle of ∽20°. Upon heating, the methyl motion speeds up and an additional reorientation of the C3 axis occurs. At the same time, the motion of the CD and CD_2_ groups becomes faster and a reorientation of the cone axis itself likely shows up. At 20 °C, corresponding to *T*_g_ − 45 °C, the motions of all the moieties are still highly spatially restricted.

^1^H FID analysis corroborates this picture, showing that at *T*_g_ − 35 °C, the dynamics of both PVB-D and PVB-H is quite far from the motional narrowing, since motions of the main-chain are slow and motions of side-chains, although faster, are quite restricted in amplitude. On increasing the temperature, a progressively faster and more isotropic dynamics is observed above *T*_g_. At the highest achieved temperature (*T*_g_ + 35 °C) a correlation time of 1.6 μs is estimated for the α-relaxation in PVB-D, while a shorter effective correlation time (1.0 μs), also accounting for the faster motions in the side-chains, results for PVB-H.

The different dynamics of main-chain and propyl side-chains is further highlighted by ^1^H FC NMR. Indeed, while the main-chain undergoes conformational reorientations associated to the α-relaxation, as detected in previous DS measurements, the side-chain moieties experience a faster secondary motion, here revealed for the first time and likely undetectable by DS. At 115 °C, the side-chains’ motion is at least 5 times faster than that of the main-chain. The β-relaxation reported in the DS investigations is outside the frequency range here investigated.

From a methodological point of view, we can state that the combination of NMR techniques and selective isotopic labeling here employed were revealed to be particularly useful for unravelling both the rate and mechanism of main- and side-chain motions in PVB. Measurements at higher temperatures are envisaged with all NMR methods in order to extend this study and further explore the motions of main- and side-chains over a broader frequency range.

## Data Availability

The data presented in this study are available on request from the corresponding author.

## Supplementary Materials

The following are available online at https://www.mdpi.com/article/10.3390/polym13162686/s1. Synthesis of PVB-H and PVB-D. Experimental and calculated ^2^H NMR spectra at −173 and −123 °C. Anisotropy and asymmetry parameters for methylene and methine deuterons as a function of temperature. ^1^H FIDs of PVB-H and PVB-D recorded by direct excitation and fittings to the Anderson-Weiss equation.
