# Supplementary material for: Unravelling Main- and Side-Chain Motions in Polymers with NMR Spectroscopy and Relaxometry: The Case of Polyvinyl Butyral
Source: Polymers (Basel). 2021 Aug 11;13(16):2686. doi: 10.3390/polym13162686 (PMC8398131; doi:10.3390/polym13162686)
Supplement: Supplementary file 1 [file polymers-13-02686-s001.zip › polymers-1285428-SI.pdf]

polymers

Supporting Information: Unravelling Main- and Side-Chain Motions in Polymers with NMR Spectroscopy and Relaxometry: The Case of Polyvinyl Butyral

Lucia Calucci, Silvia Pizzanelli, Alessandro Mandoli, Artur Birczyński, Zdzisław T. Lalowicz, Cristina De Monte, Lucia Ricci and Simona Bronco[6]

S1. Synthesis of PVB-H and PVB-D

S1.1. General Information

All the reactions involving sensitive compounds were carried out under dry N_2, in flame-dried glassware. Solvents were freshly distilled before the use from the proper drying agent [1]. If not noted otherwise, the compounds were commercially available and used as received. Polyvinyl alcohol (**4**, M_W = 31-50 kg/mol, 98-99% hydrolyzed), *N*-methyl-2-pyrrolidone (NMP), 1,2-dichlorobenzene, butanal, Dess-Martin periodinane (**2**), 37% DCl, and D_2O were purchased from Sigma-Aldrich, whereas butanol-d_{10} (**1**) was obtained from Deutero.

Unless noted otherwise, the solution-phase NMR spectra were recorded in $CDCl_3$ with a Bruker Avance DRX 400 spectrometer. Chemical shift (δ) values are reported in ppm, with frequency referencing made by setting the residual not-deuterated solvent (^{1}H) or the deuterated solvent (^{13}C) to recommended values [2].

Electron ionization mass analyses (GC-MS (EI)) were performed on a Varian Saturn 2000 spectrometer, interfaced with a Varian 3800 gas-chromatograph.

S1.2. Preparation of butanal-d_8 (3)

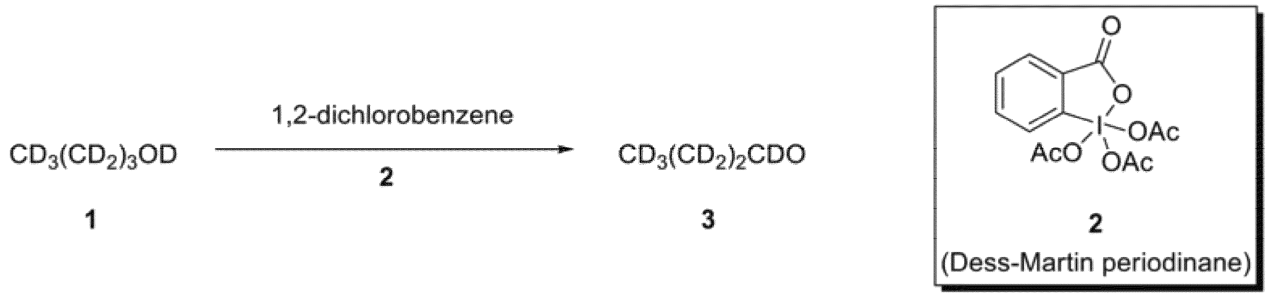

Scheme S1.

The oxidation of butanol-d_{10} (**1**) was carried out by modification of the general procedure introduced by D. B. Dess and J. C. Martin [3] according to Scheme 1.

A three-necked 250 ml flask, provided with N_2 inlet and magnetic stirring, was charged with 22.68 g (53.5 mmol) of Dess-Martin periodinane (**2**) and 80 ml of 1,2-dichlorobenzene to give a thick suspension that was stirred for 5 min at r.t. After placing the flask in a water bath, 4.14 g (49.2 mmol) of butanol-d_{10} (**1**) was added, whereupon a moderate warming of the mixture and the almost complete dissolution of the precipitate were observed within 5 min. Further stirring for 10 min led to the precipitation of a clear solid and the disappearance of **1,** as revealed by GC-MS analysis. The suspension was filtered under N_2 through a glass frit and the residue was washed with 30 ml of 1,2-dichlorobenzene. The combined filtrates, collected in a 500 ml flask, were treated with a solution of 14.5 g of $NaHCO_3$ and 6.7 g of $Na_2S_2O_3$ in 160 g of D_2O. After frothing had ceased (5 min), the layers were separated and the upper one was extracted with 3 × 20 ml of 1,2-dichlorobenzene. The combined organic extracts were dried over Na_2SO_4 before being transferred

into a 250 ml flask connected to a train of two high-efficiency condensation traps, the last of which cooled in liquid N_2. The apparatus was evacuated to approximately 1 mbar and the flask was heated with a water bath from r.t. to 55 °C to attain the evaporation of **3** within 3 h. GC-MS analysis of the condensate in the liquid N_2 cooled trap revealed the presence of **3** and 1,2-dichlorobenzene in approximately 1:1 ratio. Micro-scale fractional distillation of the mixture, through a 3 cm column packed with glass beads, afforded **3** as the forerun (54-64 °C, 1.20 g, 30% yield). The purity and the deuteration degree of the product (colorless and clear liquid) were evaluated by GC-MS (EI) and resulted >99% and >95%, respectively.

MS(EI) *m/z* (relative intensity): 50 (80), 54 (2), 60 (2), 61 (2), 62 (100), 78 (3), 80 (95), 81 (5), 82 (9).

^{13}C NMR (101 MHz, $CDCl_3$) δ 202.8 (t, J = 26.0 Hz), 44.6 (p, J = 18.4 Hz), 14.6 (p, J = 18.9 Hz), 12.6 (h, J = 19.5 Hz).

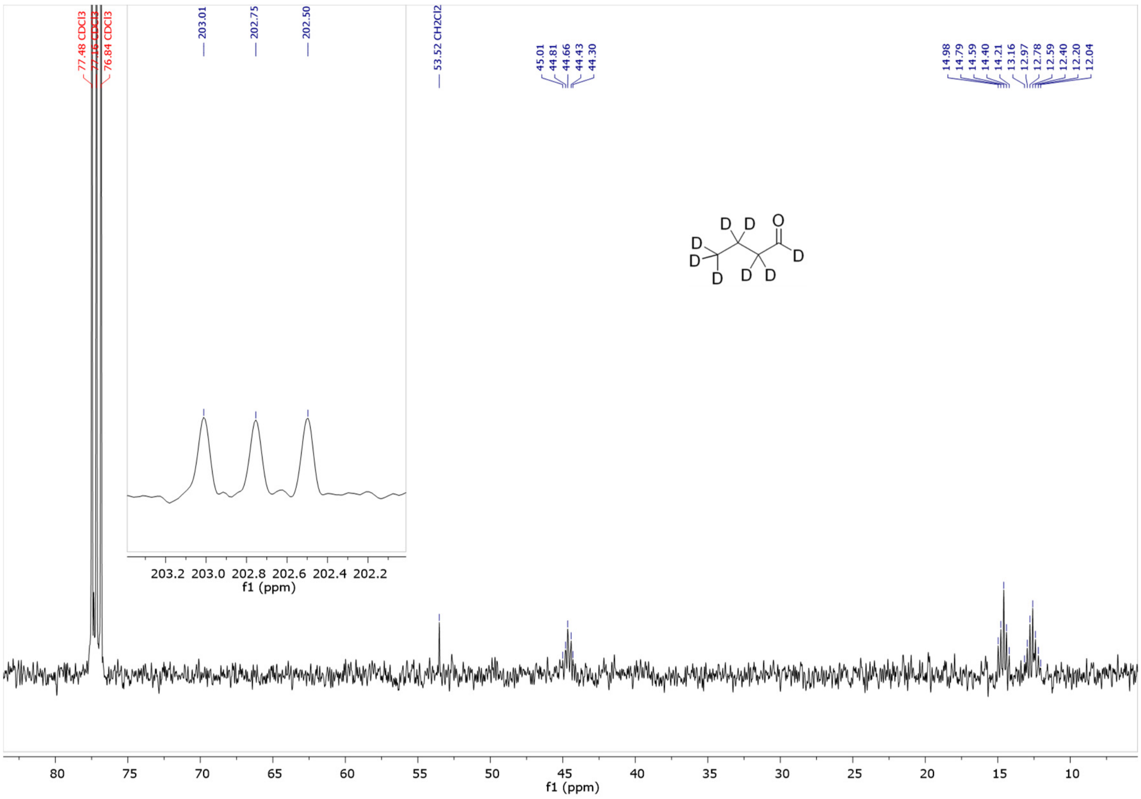

Figure S1. ^{13}C NMR spectrum of butanal-d_8 in $CDCl_3$.

S1.3. Preparation of PVB-D and PVB-H

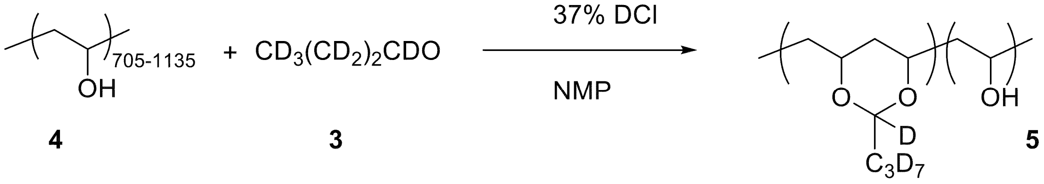

Scheme 2.

PVB-D and PVB-H were prepared following the procedure reported by Fernández et al. [4], replacing THF with acetone in the precipitation steps in order to reduce the tendency of polymer beads to stick together; the procedure is sketched in Scheme 2 for PVB-D.

For the preparation of PVB-D (**5**), a two-necked 50 ml flask, provided with N_2 inlet and magnetic stirring, was charged with 1.113 g (~ 25 mmol) of **4** and 27 ml of NMP, and

heated under stirring up to 85 °C until almost complete dissolution of the polymer beads (2 h). The solution was cooled to r.t. and treated with 0.34 ml of 37 wt% DCl in D_2O (4.1 mmol) and 0.992 g (12.4 mmol) of **3**. The flask was sealed under N_2 and kept at r.t. under stirring to give a pink opalescent solution in 30 min. After 48 h, the resulting pale-yellow solution was cannulated, followed by a 2 × 5 ml acetone rinse, into a rapidly stirred solution of 1.36 g of NaOH in 170 ml of deionized water. The white suspension was kept stirring overnight and then filtered through a medium porosity frit. The residue was washed with deionized water until neutral (4 × 100 ml) and dried under suction for 1 h. The resulting white beads were dissolved in acetone (50 ml) by stirring for 2 h, precipitated by cannulation into 250 ml of deionized water, and filtered again. The repetition of the dissolution-precipitation sequence, followed by overnight drying under suction, afforded 1.26 g of **5** as white, soft beads. Gentle magnetic stirring under vacuum (1 mbar) converted the latter into a not sticking powder, which was characterized by solution-state NMR (Figures S2 and S3).

^{1}H NMR (401 MHz, $CDCl_3$) δ 4.60 – 3.30 (m, 1H), 2.52 – 1.00 (m, 2.1H).

^{13}C NMR (101 MHz, $CDCl_3$) δ 101.2, 94.4, 73.9, 73.2, 72.6, 72.3, 43.2, 42. 8, 38.2, 37.6, 16.5, 12.9.

The repetition of the same procedure, starting from polymer **4**, butanal, and 37 wt% HCl, afforded PVB-H, which was characterized by solution-state NMR (Figures S2 and S3).

^{1}H NMR (401 MHz, $CDCl_3$) δ 5.11– 3.30 (m, 3.8H), 2.59 – 1.11 (m, 9.4H), 0.90 (br. s, 3H).

^{13}C NMR (101 MHz, $CDCl_3$) δ 101.7, 94.77, 74.0, 73.2, 72.6, 72.3, 43.1, 42.8, 37.4, 37.1, 17. 8, 17.74, 17.69, 14.1, 14.0.

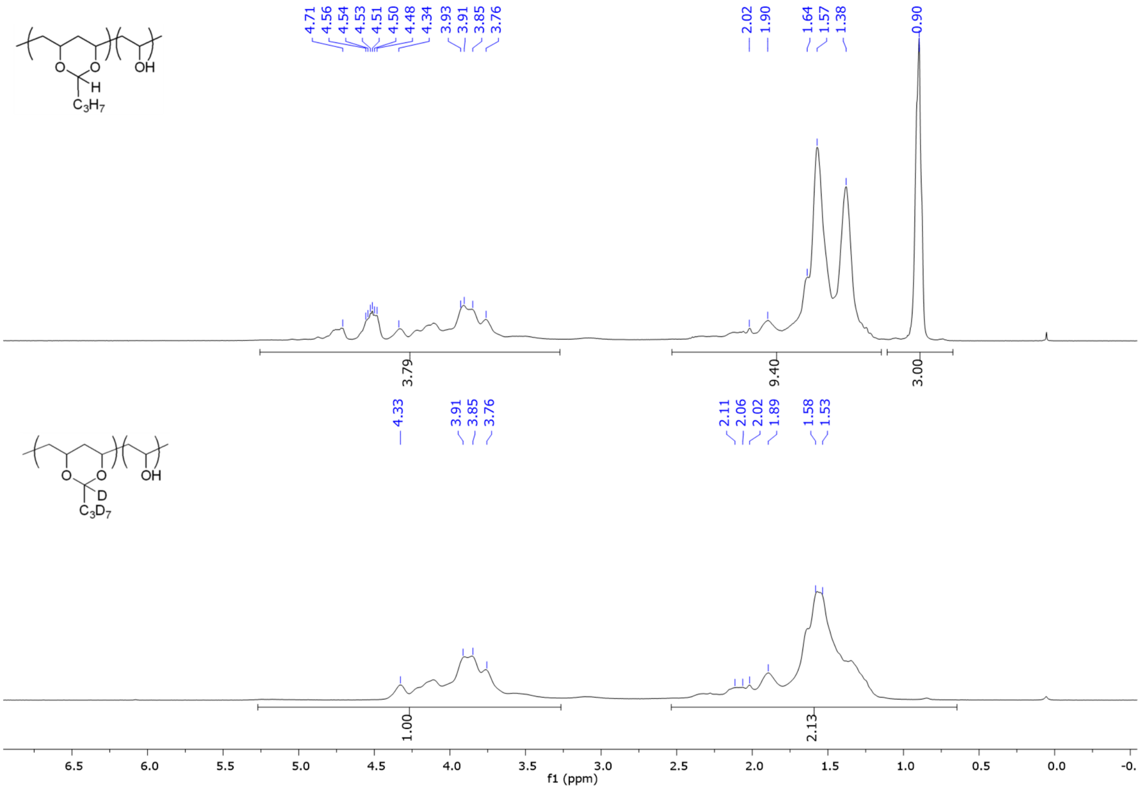

Figure S2. ^{1}H NMR spectra of PVB-D (bottom) and PVB-H (top) in $CDCl_3$.

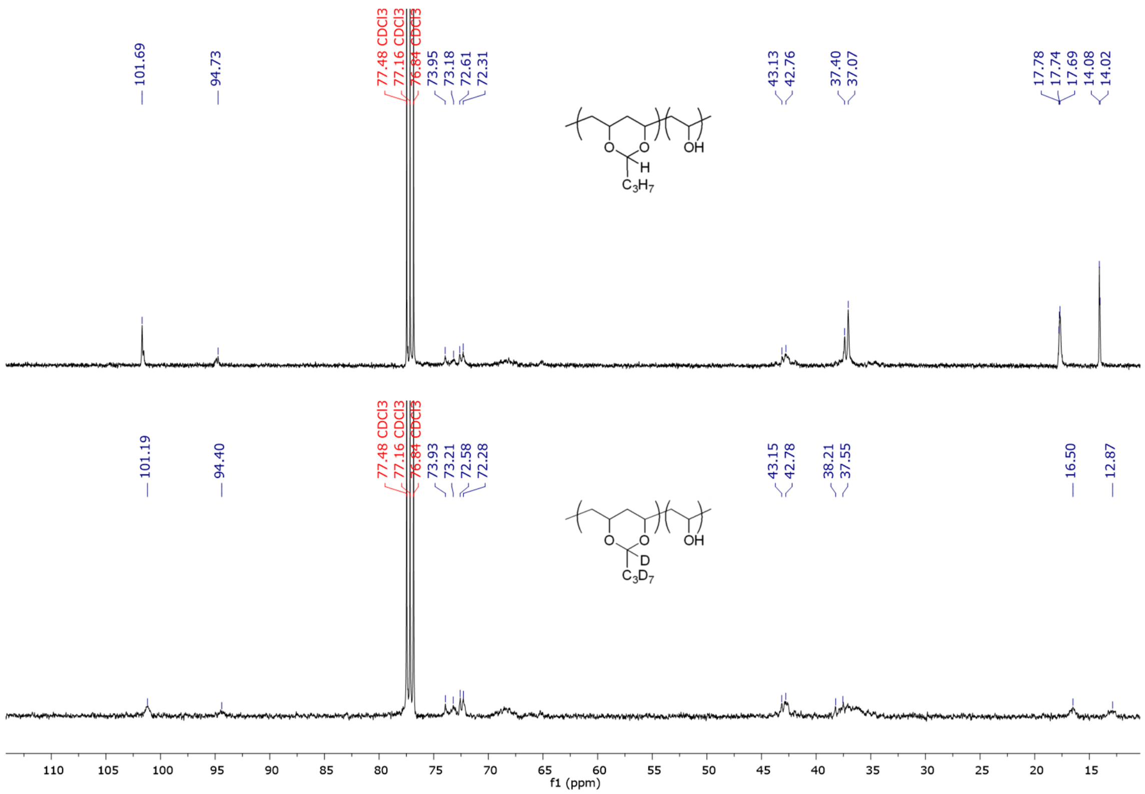

Figure S3. ^{13}C NMR spectra of PVB-D (bottom) and PVB-H (top).

S2. ^{2}H NMR spectroscopy of PVB-D

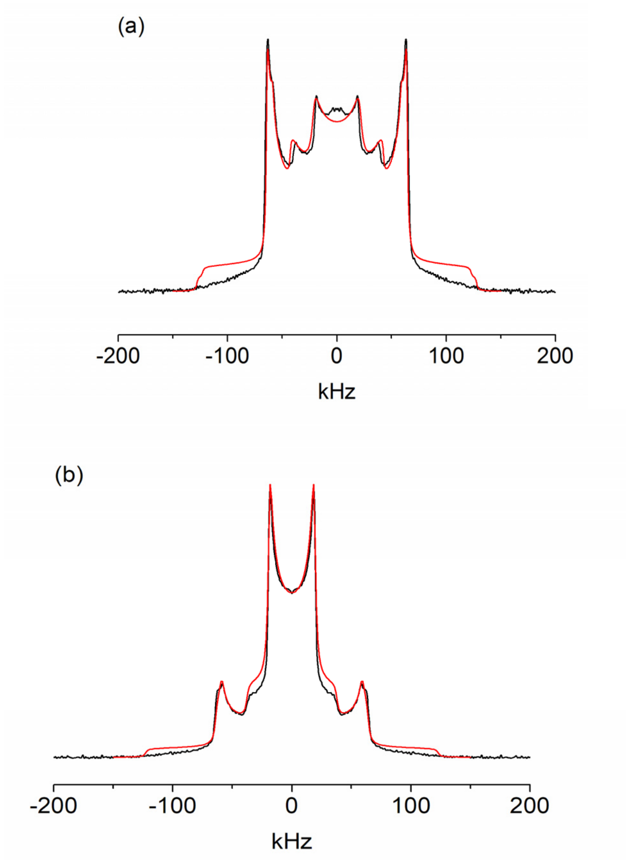

Figure S4. (a) Comparison between the experimental ^{2}H NMR spectrum of PVB-D at -173 °C (black line) and a simulated spectrum (red line) resulting from the superposition of: a rigid spectrum (42%); the spectrum of methylene and methine deuterons undergoing fast two site jumps in a cone with an

angle of 20° (28%); the spectrum of methyl deuterons subject to a three-site jump motion with a rate of 4000 kHz (30%). (b) Comparison between the experimental ^{2}H NMR spectrum of PVB-D at -123 °C (black line) and a simulated spectrum (red line) resulting from the superposition of: the spectrum of methylene and methine deuterons undergoing fast two site jumps in a cone with an angle of 20° (24%); the spectrum of methyl deuterons subject to a fast three-site jump motion (76%). The simulations were carried out using NMR Weblab [5].

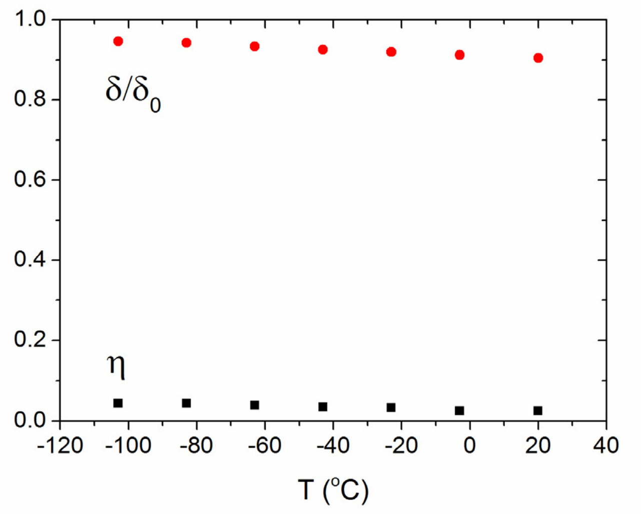

Figure S5. Normalized anisotropy (red circles) and asymmetry parameter (black squares) derived from the methylene pattern in the experimental ^{2}H NMR spectra of PVB-D at the indicated temperatures.

S3. Analysis of ^{1}H FID's of PVB-H and PVB-D

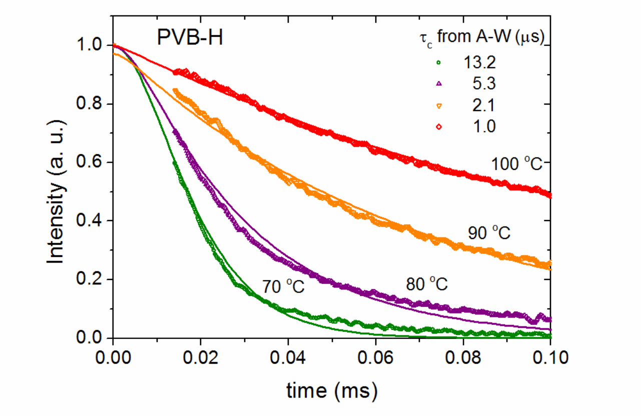

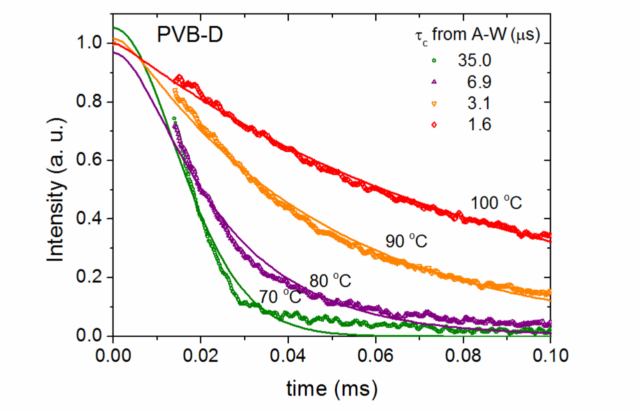

Figure S6. ^{1}H FIDs recorded on PVB-H (left) and PVB-D (right) at the indicated temperatures (symbols) by direct excitation and fitting curves to Eq. 4 of the main text (solid lines).

References

[1] Armarego, W. L. F.; Chai, C. *Purification of Laboratory Chemicals*; 5th ed.; Butterworth-Heinemann: Amsterdam, 2003.

[2] Gottlieb, H. E.; Kotlyar, V.; Nudelman, A. NMR Chemical Shifts of Common Laboratory Solvents as Trace Impurities. *J. Org. Chem*. **1997**, *62*, 7512-7515.

[3] Dess, D. B.; Martin, J. C. A Useful 12-I-5 Triacetoxyperiodinane (The Dess-Martin Periodinane) for the Selective Oxidation of Primary or Secondary Alcohols and a Variety of Related 12-I-5 Species. *J. Am. Chem. Soc*. **1991**, *113*, 7277-7287.

[4] Fernández, M. D.; Fernández, M. J.; Hoces, P. Synthesis of Poly(vinyl butyral)s in Homogeneous Phase and Their Thermal Properties. *J. Appl. Polymer Sci*. **2006**, *102*, 5007-5017.

[5] Macho, V.; Brombacher, L.; Spiess, H. W. The NMR-WEBLAB: An Internet Approach to NMR Lineshape Analysis. *Appl. Magn. Reson*. **2001**, *20*, 405-432.
